# The effect of β-carotene on the mortality of male smokers is modified by smoking and by vitamins C and E: evidence against a uniform effect of nutrient

**DOI:** 10.1017/jns.2020.3

**Published:** 2020-03-11

**Authors:** Harri Hemilä

**Affiliations:** Department of Public Health, University of Helsinki, Helsinki, Finland

**Keywords:** Antioxidants, Cohort studies, Dietary supplements, Effect modifiers, Oxidative stress, Population characteristics, Randomised controlled trials, AT, *all rac-*α-tocopheryl acetate, ATBC, Alpha-Tocopherol Beta-Carotene, BC, β-carotene, RR, risk ratio

## Abstract

A previous analysis of the Alpha-Tocopherol Beta-Carotene (ATBC) Study on male smokers found that β-carotene supplementation increased the risk of pneumonia 4-fold in those who started smoking at the age of ≥21 years and smoked ≥21 cigarettes/d (a subgroup of 7 % of the study population). The present study hypothesised that β-carotene increases mortality in the same subgroup. The ATBC Study (1985–1993) recruited 29 133 Finnish male smokers (≥5 cigarettes/d) aged 50–69 years. Cox regression models were constructed to estimate the effect of β-carotene supplementation in subgroups. β-Carotene increased mortality (risk ratio 1·56; 95 % CI 1·06, 2·3) in those who started to smoke at ≥21 years and smoked ≥21 cigarettes/d. Within this subgroup, there was strong evidence of further heterogeneity. The effect of β-carotene supplementation was further modified by dietary vitamin C intake, fruit and vegetable intake (*P* = 0·0004), and by vitamin E supplementation (*P* = 0·011). Thus, harm from β-carotene was not uniform within the study population. Interactions between β-carotene and vitamins C and E were seen only within a subgroup of 7 % of the ATBC participants, and therefore should not be extrapolated to the general population. Heterogeneity of the β-carotene effect on mortality challenges the validity of previous meta-analyses that have pooled many diverse antioxidants for one single estimate of effect using the assumption that a single estimate equally applies to all antioxidants and all people. Trial registration: ClinicalTrials.gov NCT00342992.

In 2007, Bjelakovic *et al*. published a highly cited meta-analysis on diverse antioxidants for preventing mortality^(1)^. They pooled the results of forty-seven low-bias risk trials with 180 938 participants on mortality, and calculated that antioxidants significantly increased mortality (risk ratio (RR) 1·05; *P* = 0·002). However, their meta-analysis suffers from a serious ‘apples and oranges’ problem: treatments that are too different from each other were pooled together to calculate one single estimate of effect.

To illustrate the problem of apples and oranges in the Bjelakovic meta-analysis, we may consider an analogy. If a researcher is interested in the effect of antibiotics on mortality caused by infections, and combines all diverse antibiotics into a single broad category of ‘antibiotics’, and pools all ‘antibiotic trials’ together, other researchers with basic background in clinical microbiology would consider such a project scientifically unsound, since the biology of antibiotics is very complex. It is obvious that a single universal estimate for ‘antibiotic effect on mortality’ would be meaningless.

Similarly, antioxidants are a very heterogeneous group. Vitamin C is water soluble, vitamin E is fat soluble, and Se is an inorganic element. β-Carotene (BC) is an antioxidant only under specific conditions^(2–5)^. There is no basis to consider that all antioxidants are so similar as to justify the pooling of all trials with substances belonging to the broad category of ‘antioxidants’. Analogous with the above example given for antibiotics, a single uniform 5 % estimate for ‘antioxidant effect on mortality’^(1)^ is meaningless. Thus, different antioxidants should be studied separately to calculate a specific estimate for the effect of vitamin C, another for vitamin E, and still another for BC, etc.

Furthermore, in the case of specific single antioxidants, it is unlikely that there is a uniform effect that applies to all people worldwide. Previous analyses of the large-scale Alpha-Tocopherol Beta-Carotene (ATBC) Study on 29 133 Finnish male smokers^(6–8)^ showed that the effect of vitamin E supplementation was heterogeneous for the incidence of the common cold^(9)^, pneumonia^(10–13)^, tuberculosis^(14)^, and even for total mortality^(15–17)^. Thus, there is no universal ‘vitamin E supplementation effect’ for any of these four outcomes that was applicable for all the ATBC participants. Evidently, we expect heterogeneity in vitamin E effects also to occur outside of this study, since it is not reasonable to assume that the men of the ATBC Study were fundamentally different from all other men. Furthermore, strong evidence of heterogeneity has been found for the effect of vitamin C supplementation on common cold incidence. In the general population, vitamin C has no effect^(18,19)^, but in people under heavy short-term physical stress, vitamin C halved the risk of colds^(19,20)^. Finally, there is also evidence that the effect of vitamin C on the incidence of atrial fibrillation is heterogeneous^(21)^.

Previously, the effects of BC supplementation on the common cold^(22)^ and on pneumonia^(10,23)^ were shown to be heterogeneous in the ATBC Study. The effect of BC on these two outcomes was modified simultaneously by the age at which the participant had started to smoke and by the number of cigarettes he smoked per d at the baseline of the trial. Among those men who started to smoke at the age of ≥21 years and smoked ≥21 cigarettes/d, BC increased the risk of pneumonia (RR 4·0; *P* = 0·001). However, this subgroup covered only 7 % of the ATBC participants. There was no evidence of BC influence on pneumonia for the remaining 93 % of the participants^(23)^. The age at which the participants had started to smoke and the number of cigarettes they smoked at baseline also modified the effect of BC on common cold incidence^(22)^.

The purpose of this study was to examine whether the effect of BC was heterogeneous on the total mortality of the male smokers of the ATBC Study. I hypothesised that the effect of BC on mortality would be most prominent in the same subgroup in which BC increased the risk of pneumonia by using the following reasoning. The harm of smoking is directed in particular to the lungs. Pneumonia is a disease of the lungs and, therefore, it might serve as an indicator for the conditions when the harms of BC are greatest. Pneumonia itself causes only a small proportion of total mortality. Nevertheless, the small subgroup in which BC increased the risk of pneumonia might indicate wider systemic harms, which might be expressed as increased total mortality. If there is evidence that the effect of BC on mortality is heterogeneous, the validity of calculating one single estimate of effect for all antioxidants is further undermined^(1)^.

## Experimental methods

### Participants

The design and methods of the ATBC study, which examined the effects of *all rac-*α-tocopheryl acetate (AT; 50 mg/d) and BC (20 mg/d) on the incidence of lung cancer and other cancers have been described in detail^(6–8)^. The ATBC Study is registered at the website http://www.ClinicalTrials.gov under the identifier NCT00342992. The trial was approved by the institutional review boards, and all participants gave their written informed consent.

In brief, males aged 50–69 years were eligible for inclusion if they smoked ≥5 cigarettes/d at entry and those enrolled in the trial (*n* 29 133) were randomised into one of four intervention arms and administered placebo, AT, BC, or AT + BC, using a 2 × 2 factorial design. Compliance with supplementation was high: some 90 % of the participants took more than 90 % of their prescribed capsules during their active participation in the trial; there were no differences in capsule consumption among the intervention groups^(6,7)^. Supplementation increased the serum BC level to 17-fold compared with the baseline levels^(7,24)^. The intervention continued until 30 April 1993.

### Ethics approval

This is a secondary analysis of a previously reported randomised trial^(6)^ and ethics approval is not applicable for this study.

### Baseline characteristics

Before randomisation at baseline, the men completed questionnaires on their medical and smoking histories and general background characteristics, and their weight was measured. The age at smoking initiation was missing from seven participants and they were excluded from this analysis.

At the first baseline visit, participants were given a separate, detailed dietary history questionnaire for completion at home, and the questionnaire was returned and reviewed^(25)^. The questionnaire included a sixty-three-page picture booklet with 122 photographs. Participants were asked about portion sizes for 276 common foods and mixed dishes and the usual frequency of their consumption over the previous 12 months. The dietary history questionnaire provided data regarding the intake of vitamin C, vitamin E, BC, fruit and vegetables, and alcohol consumption^(25,26)^. The validity of the dietary history questionnaire was assessed by comparing it with the food consumption records of 190 participants for twelve separate 2-d periods distributed evenly over 6 months. When participants were compared by their responses to the dietary history and food consumption questions, between 74 and 76 % of participants were found to be in either the same vitamin C intake quintile or in the within-one-quintile category^(25)^. In the reproducibility study, participants filled in the food use questionnaire three times at 3-month intervals. The intraclass correlation was 0·69 for dietary vitamin C intake in the reproducibility analysis^(25)^. Dietary data were missing from 2022 participants who were excluded from the analysis of the dietary variables. Weight was missing from seventeen participants.

### Outcome and follow-up time

Deaths were identified by using the National Death Registry, as previously described^(6,7)^. Because deaths were identified in the National Death Registry, which registers all deaths occurring in Finland, loss to follow-up was insignificant. Follow-up time for each participant began from the day of randomisation and continued until death or the end of the trial. The median follow-up time for the participants was 6·1 years, and there was a total of 169 684 person-years of observation for the 29 126 participants.

### Statistical models

Statistically significant interaction between the effects of AT and BC was previously observed in certain subgroups^(11,27)^; therefore, the present primary analysis was restricted to the no-AT participants. Thus, the first analysis was between the placebo and BC arms.

I estimated the effect of BC supplementation on mortality by using proportional hazards regression models. I calculated the RR and the 95 % CI of the RR by using the PROC PHREG procedure in SAS software (release 9.4; SAS Institute, Inc.). Regarding supplementation, the analyses followed the intention-to-treat principle. To test the statistical significance of interactions, I first added the main variables to the regression model and thereafter I added the interaction term. The statistical significance of the interaction was thereafter calculated by the likelihood ratio test. Kaplan–Meier curves were drawn according to the *survfit* procedure of the statistical software R^(28)^, and the difference between the curves was calculated with the likelihood ratio test.

The pooled effects in the forest plot were calculated by using the Mantel–Haenszel method and fixed-effect option of the *metabin* procedure of the *meta* programme of R^(28)^. In the meta-analyses, heterogeneity between the subgroups was assessed by the χ^2^ test and the *I*^2^ statistic as previously^(12)^. The *I*^2^ statistic estimates the percentage of total variation across studies that is due to true heterogeneity rather than due to chance. Values of *I*^2^ over about 50 % indicate moderate and over about 75 % indicate a high level of heterogeneity^(29)^. Two-tailed *P* values were used.

### Examination of the specificity of vitamin C in modifying the harm of β-carotene

The major sources of vitamin C in the diet of study participants were fruit and vegetables. In the ATBC Study, the total intake of fruit and vegetables was strongly correlated with the calculated vitamin C intake (*r* 0·82). Thus, it is possible that in the analysis of heavy smokers who started smoking at a later age, it is the low intake of some substance other than vitamin C intake that could explain the increased harm of BC supplementation in the low-vitamin C group. I used two approaches to evaluate the specificity of vitamin C in modifying the effect of BC supplementation.

First, I selected the same number of men with low fruit and vegetable intake, and with low BC and vitamin E intakes and this was defined as the number of participants with vitamin C intake below 90 mg/d (*n* 437). If the actual substance in fruit and vegetables that modifies the effect of BC supplementation is something other than vitamin C, a more pronounced harm would be expected in participants with a low fruit and vegetable intake. Similarly, if dietary BC or vitamin E intake level is the actual modifier of the BC supplementation effect, then a greater harm would be expected in those with low intake of those substances. The cut-off limits were 90 mg/d, 146·7 g/d, 1·621 mg/d, and 10·7 mg/d for vitamin C, fruit and vegetable, BC, and vitamin E intakes, respectively, so that the group with lower intake consisted of 437 participants in each case.

Second, I calculated the residual of fruit and vegetable intake by using linear regression to model the total of fruit and vegetables as a function of dietary vitamin C, as previously described^(15,30)^. As designed, the residual of fruit and vegetable intake has no correlation with vitamin C intake. If we assume that any other putative substance(s) that might modify the effect of BC supplementation has no perfect correlation with vitamin C, then the variation in the other substance(s) remains as a variation in the residual of fruit and vegetables. A low residual of fruit and vegetables indicates less than average intake of these food classes, whereas a high residual indicates more than the average quantity of fruit and vegetables. Thus, the residual value does not indicate an absolute level of intake, but instead it indicates a relative intake level compared with the average fruit and vegetable intake for any given vitamin C intake level. Calculation of the residual adjusts for the variation in vitamin C intake so that there is little difference in vitamin C intake in the low *v.* high fruit and vegetable residual groups. In the comparison of the low and high residual fruit and vegetable intakes, the residual intake was split at the median residual of −2·4 g/d.

## Results

The ATBC Study used a 2 × 2 factorial design so that half of participants were given BC and the other half were not. Similarly, half the participants were given vitamin E and the other half were not^(6,7)^. In certain subgroups^(11,27)^, statistically significant interactions between BC and vitamin E were observed, and, therefore, the first analysis conducted was between the placebo and BC arms.

**Fig. 1.**
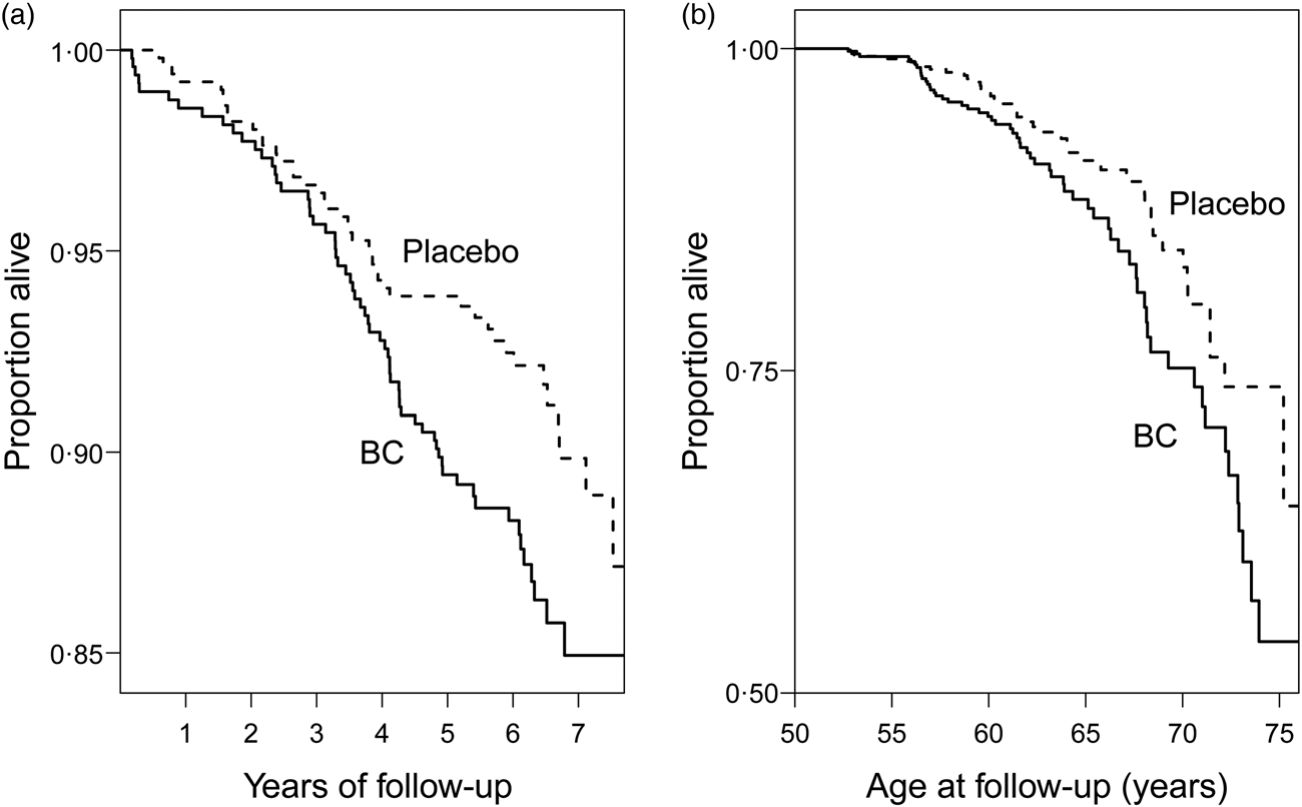
Mortality by β-carotene (BC) supplementation: participants who initiated smoking at ≥21 years and smoked ≥21 cigarettes/d (Alpha-Tocopherol Beta-Carotene Study 1985–1993). The Kaplan–Meier survival curves for the BC and placebo arms are shown. Each step indicates one death. (a) Mortality after randomisation. For the difference between the two curves, *P* = 0·024. (b) Mortality by follow-up age.

BC-supplemented participants had a higher mortality in the 14 564 men who were not administered vitamin E (RR 1·088; 95 % CI 0·99, 1·20; *P* = 0·08). There were 920 deaths in the BC arm and 850 deaths in the placebo arm. BC increased mortality significantly (RR 1·56) among the 990 participants who had initiated smoking at the age of ≥21 years and smoked ≥21 cigarettes/d at the study baseline (Table 1). In this subgroup of heavy smokers who started smoking at a later age, the Kaplan–Meier curves for the mortality of the BC and placebo arms diverge significantly (Fig. 1). Divergence started soon after randomisation and the arms remain diverged over the follow-up time (Fig. 1(a)). Mortality increases with age and the difference between the placebo and the BC arms was most prominent at the upper age range (Fig. 1(b)).

**Table 1.** Modification of the effect of β-carotene on mortality by the level of smoking and the age of smoking initiation (Alpha-Tocopherol Beta-Carotene Study 1985–1993)* (Numbers of participants; risk ratios (RR) and 95 % confidence intervals)

Smoking at study baseline (cigarettes/d)	Age of smoking initiation							
	≤20 years (median 18 years)				≥21 years (median 24 years)			
	Placebo	β-Carotene	RR	95 % CI	Placebo	β-Carotene	RR	95 % CI
5–20 (median 16)								
Deaths	428	445	1·03	0·90, 1·18	147	165	1·12	0·90, 1·40
Participants	3550	3555			1336	1365		
21–90 (median 30)								
Deaths	232	248	1·09	0·91, 1·30	43	62	1·56	1·06, 2·3
Participants	1892	1876			506	484		

* This Table is restricted to the 14 564 no-vitamin E participants. Adding a uniform β-carotene effect gave a RR of 1·088 (95 % CI 0·99, 1·20). Adding an individual β-carotene effect, shown in this Table, to each of the four subgroups non-significantly improved the fit of the Cox regression model (χ^2^ (3 df) = 4·2; *P* = 0·24). Within the subgroup of the heavy smokers (≥21/d) who started smoking late in their life (≥21 years), in the lower right-hand corner, the difference between the β-carotene and placebo arms was significant (*P* = 0·024; see Fig. 1).

**Fig. 2.**
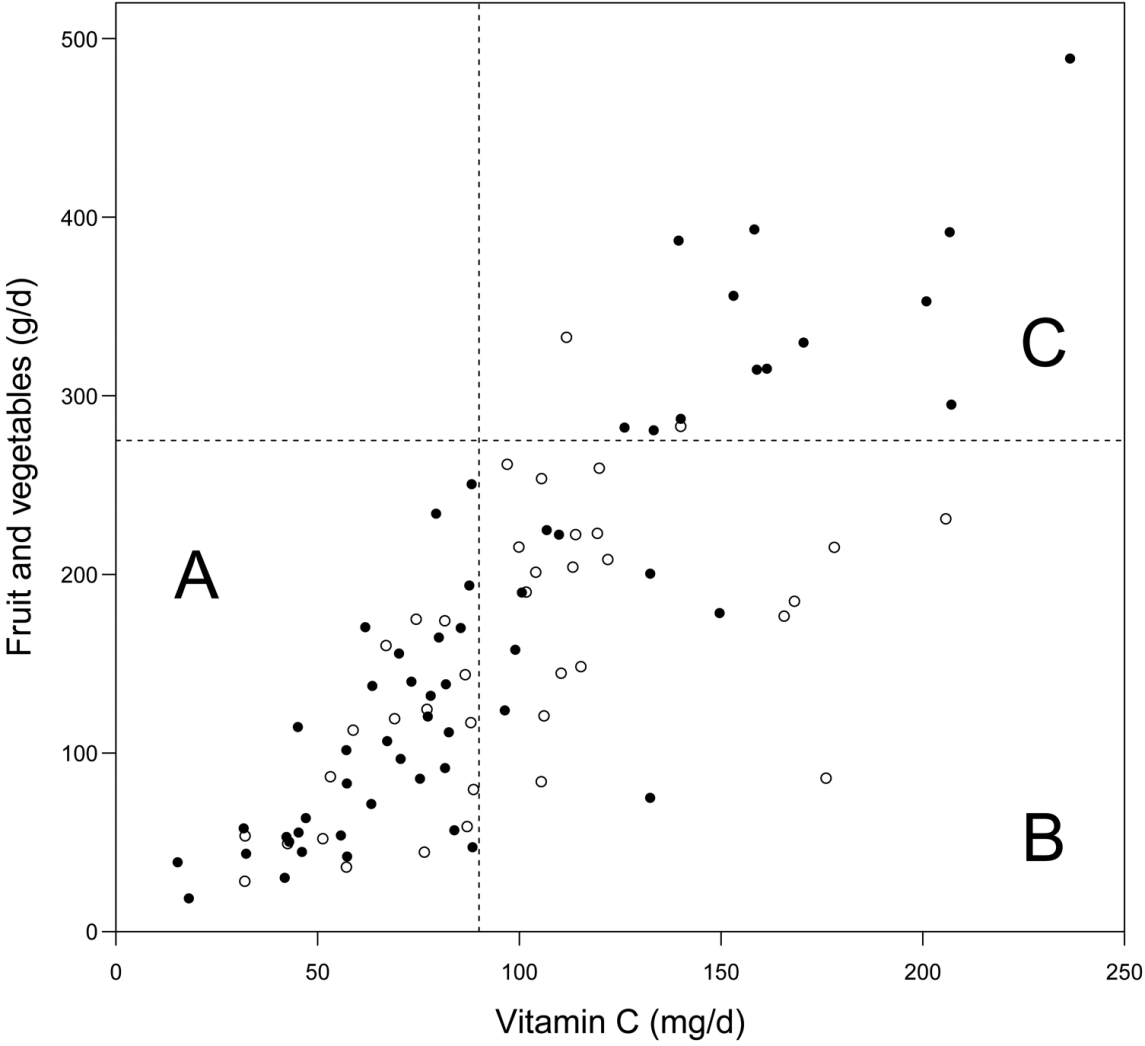
Effect of vitamin C intake, and fruit and vegetable intake on the mortality caused by β-carotene (BC) supplementation: participants who initiated smoking at ≥21 years and smoked ≥21 cigarettes/d in male smokers (Alpha-Tocopherol Beta-Carotene Study 1985–1993). ○, Deaths in the placebo arm; ●, deaths in the BC arm. Assuming that BC had no effect on mortality, we would expect a similar distribution of deaths in the placebo and BC arms. The cut-off limits used in the statistical analysis are shown: 90 mg/d vitamin C and 275 g/d fruit and vegetables. The subgroup labels A, B and C are as used in Table 3.

**Fig. 3.**
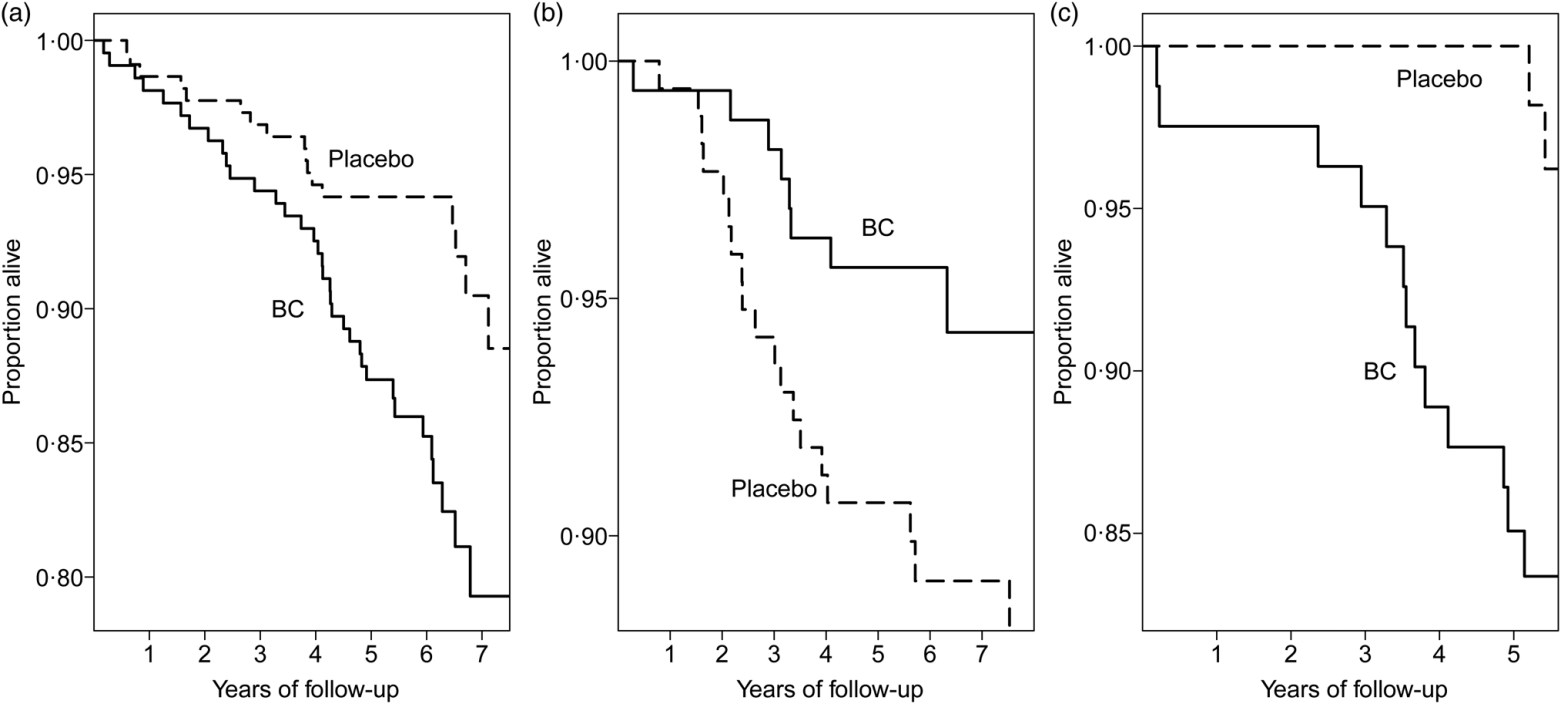
Mortality caused by β-carotene (BC) supplementation in subgroups A to C of Fig. 2 in male smokers (Alpha-Tocopherol Beta-Carotene Study 1985–1993). The Kaplan–Meier survival curves are shown for the BC and placebo arms. Each step indicates one death. (a) Subgroup A: vitamin C < 90 mg/d. For the difference between the two arms, *P* = 0·004. (b) Subgroup B: vitamin C ≥90 mg/d and fruit and vegetables <275 g/d, *P* = 0·049. (c) Subgroup C: vitamin C ≥90 mg/d and fruit and vegetable intake ≥275 g/d, *P* = 0·004.

The deaths among the heavy smokers who started smoking at a later age are plotted by the dietary intake of vitamin C and by the intake of fruit and vegetables in Fig. 2. It appears that the ratio of deaths in the BC arm *v.* placebo arm is higher on the lower-left hand side and on the upper-right hand side. In participants who had vitamin C intake <90 mg/d, BC supplementation increased mortality (RR 2·26; 95 % CI 1·27, 4·0; subgroup A). For those who had vitamin C intake ≥90 mg/d and fruit and vegetable intake ≥275 g/d, BC increased mortality (RR 6·1; 95 % CI 1·4, 27; subgroup C). For those who had vitamin C intake ≥90 mg/d, but fruit and vegetable intake <275 g/d, there was no evidence of harm from BC (subgroup B). The Kaplan–Meier curves of the three subgroups are shown in Fig. 3.

Since the cut-off limits in Fig. 2 were decided after evaluating those data, the interaction between the BC and continuous diet variables was also calculated. When restricting to fruit and vegetable intake <275 g/d, there was a significant interaction between BC supplementation and dietary vitamin C intake (*P* = 0·0004). When restricting to vitamin C intake ≥90 mg/d, there was significant interaction between BC supplementation and fruit and vegetable intake (*P* = 0·042).

Participants with vitamin C intake <90 mg/d also had low intakes of many other substances of fruit and vegetables so that the factor that modified the BC supplementation effect in subgroup A of Fig. 2 might be something other than vitamin C. First, the same number (*n* 437) of men was selected who had the lowest intake of fruit and vegetables, BC, and vitamin E, and the effect of BC supplementation was RR 1·8 (95 % CI 1·00, 3·2; *P* = 0·045), 1·5 (95 % CI 0·86, 2·7) and 1·8 (95 % CI 1·03, 3·2; *P* = 0·037), respectively. Thus, none of these variables better captured the harm of BC supplementation than for the low vitamin C intake subgroup (RR 2·26).

A second approach to evaluate the specificity of vitamin C in modifying the effect of BC supplementation was by calculating the residual fruit and vegetable intake in the heavy smokers (≥21/d) who started smoking late in life (≥21 years), and had fruit and vegetable intakes <275 g/d. In the upper part of Table 2, the participants are divided by the median intake of vitamin C intake. There was significant heterogeneity in the BC effect between those who had low and high dietary vitamin C intakes (*P* = 0·0014). However, that difference could be caused by substances other than vitamin C that are also present in fruit and vegetables.

**Table 2.** Examination of the specificity of vitamin C in modifying the effect of β-carotene (BC) supplementation in male smokers (Alpha-Tocopherol Beta-Carotene Study 1985–1993)*

Variable	Subgroup division			
Vitamin C	Below median (84 mg/d)		Above median (84 mg/d)		
	Placebo	BC	Placebo	BC	Test of interaction: *P*
Deaths	13	31	23	12	
*n*	189	192	202	178	
Person-years	1149	1090	1186	1078	
Rate (1/1000 person-years)	11·3	28·4	19·4	11·1	
RR		2·58		0·58	0·0014
95 % CI		1·35, 4·90		0·29, 1·17	
Mean vitamin C (mg/d)	60		114		
Mean fruit and vegetables (g/d)	94		180		
Residual of fruit and vegetables	Below median (−2·4 g/d)		Above median (−2·4 g/d)		
	Placebo	BC	Placebo	BC	
Deaths	19	25	17	18	
*n*	198	182	193	188	
Person-years	1170	1043	1165	1124	
Rate (1/1000 person-years)	16·2	24·0	14·6	16·0	
RR		1·51		1·10	0·5
95 % CI		0·83, 2·80		0·57, 2·13	
Mean vitamin C (mg/d)	82		92		
Mean fruit and vegetables (g/d)	92		182		

RR, risk ratio.

* This Table is restricted to the heavy smokers (≥21/d) who started smoking late in life (≥21 years), and had fruit and vegetable intakes <275 g/d.

The lower part of Table 2 shows the comparison over the residual fruit and vegetable intake (see Methods). In the lower part, the 2-fold variation in fruit and vegetable intake remains, as does the variation in substances that are not closely correlated with vitamin C. However, the difference in vitamin C intake is minimal between the two groups. There is no evidence that the BC supplementation effect differs by the 2-fold fruit and vegetable intake variation (*P* = 0·5). The two approaches indicate that vitamin C may be the specific substance that modifies the effect of BC supplementation.

The same subgroups A to C that are shown in Fig. 2 are also shown in Table 3. In addition to the BC and placebo arms, this table also includes the vitamin E (AT) and BC + vitamin E (AT + BC) arms. Allowing the nine treatment effects in Table 3 leads to a significant improvement in the statistical model (*P* = 0·009), when compared with the model that assumes no effects of BC or vitamin E. This improvement indicates that either BC or vitamin E, or a combination of both have genuine treatment effects in some cells of Table 3. Thereafter the heterogeneity of the BC, AT, and AT + BC effects were studied in the table. Compared with the placebo arm, there is highly significant heterogeneity in the BC arm data amongst the three subgroups of A to C, which are categorised by the intake of vitamin C and fruit and vegetables (*P* = 0·0004), but no evidence of heterogeneity in the AT and AT + BC arms. Finally, there was also a significant interaction between AT and BC over the three subgroups A to C (*P* = 0·011).

**Table 3.** Effects of β-carotene alone (BC), α-tocopherol alone (AT) and α-tocopherol and β-carotene combined (AT + BC) on mortality by vitamin C intake, and by fruit and vegetable intake in heavy smokers who started smoking late in their life (Alpha-Tocopherol Beta-Carotene Study 1985–1993)*

Subgroup	Placebo	BC	AT	AT + BC	Test of interaction: *P*
Group C: vitamin C ≥ 90 mg/d and fruit and vegetables ≥275 g/d					
RR	1·0	6·1	2·8	2·5	0·030
95 % CI	Reference	1·4, 27	0·57, 14	0·48, 13	
Deaths	2	13	6	5	
Participants	69	81	78	73	
Person-years	429	469	468	443	
Rate	4·7	27·7	12·8	11·6	

Group B: vitamin C ≥ 90 mg/d and fruit and vegetables <275 g/d					
RR	1·0	0·45	0·85	0·98	0·08
95 % CI	Reference	0·19, 1·02	0·44, 1·7	0·51, 1·9	
Deaths	19	8	16	17	
Participants	171	157	168	157	
Person-years	1010	945	995	915	
Rate	18·8	8·5	16·1	18·6	

Group A: vitamin C < 90 mg/d					
RR	1·0	2·26	1·50	1·63	0·06
95 % CI	Reference	1·27, 4·00	0·82, 2·70	0·90, 3·00	
Deaths	17	35	29	30	
Participants	223	214	261	251	
Person-years	1341	1228	1532	1463	
Rate	12·7	28·5	18·9	20·5	
Test of difference (2 df) over the subgroups A to C; *P*		0·0004	0·3	0·4	

RR, risk ratio.

* This subgroup analysis is restricted to the heavy smokers (≥21/d) who started smoking late in life (≥21 years), i.e. the lower right-hand corner in Table 1. In all, 145 participants with missing dietary data were excluded from this analysis, which left 1903 participants. A conservative approach to analyse this Table is to first assume that each subgroup A to C has a uniform mortality rate over all the trial arms. Thereafter each of the three treatment groups BC, AT and AT + BC in each of the three subgroups A to C are allowed their own treatment effect, leading to a total of 3 × 3 = 9 effect estimates. When the nine treatment effects were allowed for the Cox model, it was improved (χ^2^ (9 df) = 22·0; *P* = 0·009), compared with the model that assumes no effects of BC or vitamin E or their combination. Thereafter, the heterogeneity within the treatment arms was further examined. Adding a uniform BC effect gave RR 1·56 (95 % CI 1·03, 2·36), a uniform vitamin E (AT) effect gave RR 1·25 (95 % CI 0·82, 1·90), and a uniform AT + BC effect gave RR 1·37 (95 % CI 0·90, 2·08). When individual BC effects shown in this Table were allowed for the three subgroups A to C in the BC arm, the Cox regression model was improved (χ^2^ (2 df) = 15·7; *P* = 0·0004). When individual AT effects were allowed for the three subgroups of the AT arm, the model was improved (χ^2^ (2 df) = 2·8; *P* = 0·3), and individual AT + BC effects to the AT + BC arm improved the model (χ^2^ (2 df) = 1·8; *P* = 0·4). The interaction between BC and vitamin E in subgroups A to C in this Table was calculated by first including the BC and vitamin E effects into the model, and thereafter adding the interaction terms. The fit of the Cox model increased (χ^2^ (3 df) = 11·1; *P* = 0·011). There are no substantial baseline differences between the four arms in subgroups A, B or C (Supplementary Table S1 of Supplementary material).

Given that BC and vitamin E have a statistically significant interaction in Table 3, the BC and AT + BC arms were also compared head-to-head to determine whether vitamin E reverses the effects of BC (Table 4). Over the three subgroups, the combination of BC and vitamin E differed significantly from BC alone. Table 3 shows that vitamin E has no effect over the placebo arm; thus, the AT + BC effect shown in Table 4 data is a reversion of the harm from BC alone.

**Table 4.** Prevention of the harms from β-carotene by vitamin E (Alpha-Tocopherol Beta-Carotene Study 1985–1993)*

Subgroup	BC	AT + BC
Group C: vitamin C ≥90 mg/d and fruit and vegetables ≥275 g/d		
RR	1·0	0·39
95 % CI	Reference	0·14, 1·09
Deaths	13	5
Participants	81	73
Person-years	469	443
Rate	29·1	11·6

Group B: vitamin C ≥90 mg/d and fruit and vegetables <275 g/d		
RR	1·0	2·2
95 % CI	Reference	0·95, 5·1
Deaths	8	17
Participants	157	157
Person-years	945	915
Rate	8·3	18·4
		
Group A: vitamin C < 90 mg/d		
RR	1·0	0·72
95 % CI	Reference	0·44, 1·2
Deaths	35	30
Participants	214	251
Person-years	1228	1463
Rate	28·5	20·5
Test of difference (2 df) over the subgroups A to C; *P*		0·017

BC, β-carotene alone; AT + BC, α-tocopherol and β-carotene combined; RR, risk ratio.

* This Table shows the head-to-head comparison of the BC and the AT + BC arms shown in Table 3. The BC arm is used as the reference level in this comparison, since the focus is on the effects of vitamin E above BC. Adding a uniform AT + BC effect gave RR 0·87 (95 % CI 0·59, 1·27). Adding an individual AT + BC effect, shown in this Table, to each of the three subgroups improved the fit of the Cox regression model (χ^2^ (2 df) = 8·1; *P* = 0·017).

The dose of BC in the ATBC Study was fixed and, therefore, dose–response cannot be examined by variation over the dosage *per se*. However, the weights of the men varied, which entails that a constant dose for a light-weight person corresponds to a higher dose per unit weight, compared with a heavy-weight person. In subgroup A, there was significant modification of BC effect by weight. The harm of BC was restricted to participants who had low body weights. No evidence of harm was seen in those who had high body weights (Table 5). The harm was also most evident in those who consumed more alcohol, but the difference between the alcohol intake subgroups was not significant.

**Table 5.** Effect of β-carotene (BC) on mortality by weight and alcohol intake in subgroup A (Alpha-Tocopherol Beta-Carotene Study 1985–1993)*

Subgroup	Placebo	BC
All		
RR	1·0	2·29
95 % CI	Reference	1·28, 4·1
Deaths	17	35
Participants	223	214

Weight		
<80 kg		
RR	1·0	4·4
95 % CI	Reference	1·8, 11
*P*		0·0003
Deaths	6	24
Participants	121	117
≥80 kg		
RR	1·0	1·1
95 % CI	Reference	0·49, 2·6
*P*		0·8
Deaths	11	11
Participants	102	97

Alcohol		
<14 g/d		
RR	1·0	1·6
95 % CI	Reference	0·8, 3·5
*P*		0·2
Deaths	12	16
Participants	113	98
≥14 g/d		
RR	1·0	3·9
95 % CI	Reference	1·4, 11
*P*		0·003
Deaths	5	19
Participants	110	116

RR, risk ratio.

* This Table is limited to the placebo and BC arms of subgroup A of Table 3. The test of interaction between body weight and BC gives *P* = 0·02, and between alcohol and β-carotene gives *P* = 0·15.

Subgroups A to C consist of small proportions of the entire ATBC cohort, 3 % or less of all the participants in each group, which amounted to a total of 7 % of the entire study population of the ATBC Study. However, since the ATBC Study was very large, these three subgroups are sufficiently large in themselves so that there are no substantial baseline differences between the four treatment arms on age, weight, level of smoking, age of smoking initiation, intake of vitamin C, BC, fruit, vegetables, or alcohol (Supplementary Table S1 in Supplementary materials). Thus, the highly significant heterogeneity between the placebo and BC arms in Table 3 cannot be explained by substantial random baseline variations caused by restriction to small samples of the ATBC cohort.

Given that the harm of BC was greatest in those who smoked the most heavily, I also analysed the effect of BC supplementation on those who smoked the least, only 5–19 cigarettes/d among the participants who were not administered vitamin E. The median and mean level of smoking was twelve cigarettes per d in this group. There was no evidence of any harm of BC for 5309 participants (RR 0·98; 95 % CI 0·84, 1·15), based on 314 and 313 deaths in the placebo and BC arms, respectively. Within this group, there was no modification of BC supplementation effect by dietary vitamin C, fruit and vegetables, weight or alcohol intake (Supplementary Table S2 of Supplementary material).

**Fig. 4.**
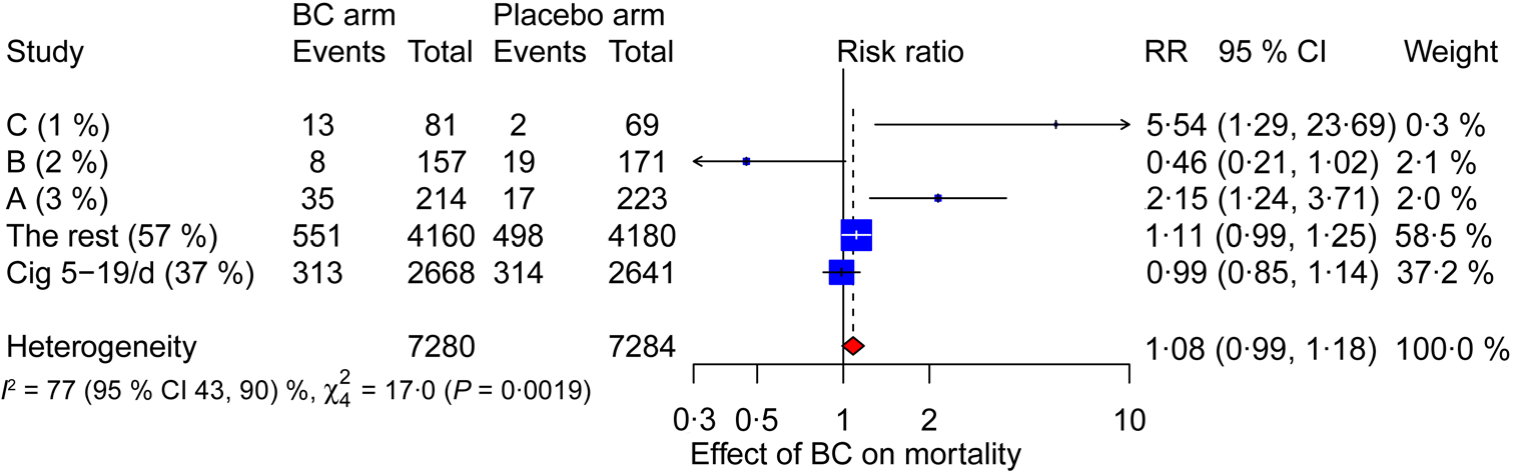
Meta-analysis to examine heterogeneity in the effects of β-carotene (BC) on mortality within the Alpha-Tocopherol Beta-Carotene Study. This meta-analysis is restricted to the 14 564 no-vitamin E participants. Subgroups A, B and C are described in Fig. 2 and Table 3. The participants who smoked between 5 and 19 cigarettes (Cig)/d (median 12/d), are separated into their own subgroup. ‘The rest’ means all the remaining participants. The percentage after the study label indicates the proportion of all participants in the particular subgroup. The horizontal lines indicate the 95 % CI for the BC effect and the squares in the middle of the horizontal lines indicate the point estimates of the effect in the particular subgroup. The diamond shape indicates the pooled effect and its 95 % confidence interval. RR, risk ratio.

The heterogeneity of the BC supplementation effect is also illustrated with a forest plot (Fig. 4). When the three subgroups A to C, and the least smoking participants, and the rest of the ATBC participants are pooled in a meta-analysis, there is high level of heterogeneity over the five subgroups (*I*^2^ = 77 (95 % CI 43, 90) %; *P* = 0·002).

## Discussion

### Three large trials on β-carotene in the 1990s

Interest in the possible health benefits of BC increased in the 1980s. Peto *et al*. summarised biological and epidemiological studies, which indicated that BC might prevent cancer^(31)^. Burton & Ingold reported that BC appeared to function as an antioxidant at low oxygen levels, whereas it appeared to function as a pro-oxidant at high oxygen levels^(2)^. Because of the enthusiasm towards BC, three large randomised trials were initiated to test whether BC protects against lung cancer. They were all published in the middle of the 1990s.

The ATBC Study examined 29 133 male smokers^(6–8,32)^, the Beta-Carotene and Retinol Efficacy Trial (CARET) 18 314 male and female smokers and asbestos workers^(33–35)^, and the Physicians' Health Study 22 071 male physicians^(36,37)^. The first two trials unexpectedly found that BC increased the risk of lung cancer and total mortality, whereas the third found no effects of BC. This led to an extensive re-evaluation of the potential health effects of BC^(3,38–51)^. Although these three large trials refuted any putative benefits of BC supplementation, the characteristics of people who get harm are still poorly understood^(52,53)^. This question is relevant for a better understanding of the physiology of BC in humans, i.e. what explains the observed harms, and also for public health since the specific groups of people who get the greatest harm, in particular, could be instructed to avoid high-dose BC supplementation.

### Heterogeneity in the effects of β-carotene on mortality

Based on the previous evidence of heterogeneity in the effects of BC on the incidence of the common cold and pneumonia in the ATBC Study^(10,22,23)^, I hypothesised that the effects of BC on mortality would be restricted to, or would be most prominent in those who smoked the most and had started to smoke at a later age. The distribution in BC effect on mortality was as expected (Table 1). There was a 56 % increase in mortality in the same subgroup in which BC increased the risk of pneumonia by 4-fold^(23)^. On the other hand, BC had no effect on mortality in the no-vitamin E participants who smoked just 5–19 cigarettes/d, consistent with the previous analysis on lung cancer in the ATBC Study^(8)^, and with the study on physicians who did not smoke much^(36,37)^.

One of the variables defining the subgroup in which BC was most harmful was smoking heavily at the start of the trial (Table 1). Smoking modifies BC metabolism, which leads to decreases in plasma BC levels^(54–58)^, and quitting smoking increases BC levels^(59)^. In model systems, cigarette smoke exposure has led to the degradation of BC into numerous cleavage products^(60–69)^. Oxidation of BC generates, for example, epoxides and aldehydes. There is much evidence that indicates that some of the BC breakdown products can have various harmful effects, for example, some were found to interfere with retinoid signalling ^(70–98)^. Thus, BC reacts with oxidants and therefore it has been considered as an antioxidant by many people. However, there is little, if any, evidence that it is a physiologically relevant antioxidant^(3,4,40)^. Instead, many of the oxidation products of BC appear to be harmful. These findings can explain why high-dose BC supplementation is harmful for heavy smokers in particular.

Another variable that defines the subgroup of the greatest harm was the age of smoking initiation. The age of smoking initiation was cut at 21 years in Table 1. However, this is a pragmatic cut-off limit and should not be interpreted as an accurate biological limit. Rather, a more robust characterisation of the groups is by the median ages, i.e. 18 *v.* 24 years as the age of starting to smoke. Smoking causes dozens of epigenetic changes^(99,100)^, and starting to smoke at a younger age might influence the developmental processes of the lungs differently than starting to smoke at a later age. Other studies have indicated that smoking in adolescence impairs lung growth and may lead to lower levels of maximally attained lung function^(101–103)^. Thus, initiating smoking in adolescence may cause permanent changes in the lungs, which may explain the differences in the effects of high-dose BC supplementation decades later when it is evaluated by the age when the person had started to smoke.

### Interaction of β-carotene with vitamin C, vitamin E, and fruit and vegetable intake

As a fully novel finding in this study, within the subgroup of heavy smokers who started smoking at later age, the harm of BC was found to be significantly modified by the intake of vitamin C and by the total intake of fruit and vegetables. A few *in vitro* studies have indicated that vitamin C may protect BC from oxidation^(104–109)^. Therefore, the harm of BC was expected to be most prominent in males who had low dietary vitamin C intake. Consistent with this hypothesis, increased harm of BC was indeed observed in subgroup A of the BC arm (Fig. 2; Table 3). This effect of low vitamin C intake was not explained by low fruit and vegetable intake, nor by low dietary intakes of BC or vitamin E.

Based on the above reasoning, less or no harm was expected in participants who had high dietary vitamin C intake. Unexpectedly, at high vitamin C intakes, BC harm was found to be modified by the total fruit and vegetable intake (Fig. 2; Table 3). When fruit and vegetable intake was low, BC supplementation had no effect on mortality (subgroup B), but when their intake was high, BC increased mortality significantly (subgroup C). There is no clear explanation for this modification by fruit and vegetable intake. It is possible that high doses of phytochemicals in large amounts of fruit and vegetables might compete for the metabolism of high-dose BC^(3,110)^, so that decreased rates of BC catabolism might lead to higher levels of non-physiological oxidation products in the heavy smokers. Although the biological explanation for the harm in subgroup C is speculative, the test of difference between the placebo and the BC arms in subgroup C is highly significant (Fig. 3(c)), which indicates true harm of BC in this subgroup.

A few *in vitro* studies have indicated that vitamin E may protect BC from oxidation^(4,60,109,111–114)^. In addition, clinical-level interaction between vitamin E and BC was found in certain subgroups of the ATBC study^(11,27)^. Therefore, the AT and AT + BC arms were included in the analysis of the heavy smokers who started smoking at a later age. BC and vitamin E had significant interactions in subgroup C, and also over all the three groups (Table 3). In addition, administration of vitamin E prevented the harm of BC in subgroups A and C (Tables 3 and 4).

Even when allowing each of the three subgroups in the three treatment arms of Table 3 to have an independent treatment effect, there was highly significant evidence of heterogeneity over the BC, AT, and AT + BC arms. Although there is very strong evidence to indicate that vitamin C and fruit and vegetables, and vitamin E modify the harm of BC administration in this study, it must be emphasised that this interaction was seen in only a small subgroup (7 % of the ATBC Study participants). Furthermore, the dose of BC was very high in the ATBC Study as it led to a 17-fold increase in the plasma BC level^(7,24)^. Therefore, the observed interactions may not have practical importance in the general population.

Further possible heterogeneity by weight and alcohol intake was analysed with the following reasoning. Given a fixed dose of BC, a lower weight leads to a greater dose per weight ratio and might lead to a greater harm of BC, and previously weight appeared to modify the effect of vitamin E on pneumonia^(115)^. Alcohol influences the metabolism of BC^(116)^ and alcohol appeared to modify the effect of BC on lung cancer^(8)^. These two variables may modify the effect of BC on mortality in subgroup A (Table 4).

### Problems in the meta-analysis by Bjelakovic *et al*.

When there is evidence of heterogeneity in the effects between antioxidants, and even within single antioxidants, it is scientifically unsound to calculate one single estimate of a 5 % increase in mortality, and give the impression that such an estimate would be equally meaningful for all antioxidants and for all people^(1)^.

A fundamentally important goal in modern biomedicine is specificity. The strength of a randomised trial is that the difference between the trial groups can be specifically attributed to the intervention that was tested. However, when the interventions included by Bjelakovic *et al*. varied from single antioxidants to various combinations of diverse antioxidants, so that ‘in eleven trials participants were supplemented with different mixtures of antioxidants as well as with vitamins and minerals without antioxidant properties'^(1)^, we lose specificity because of the apples and oranges problem. Bjelakovic *et al*. should have known that there was strong evidence that the vitamin E effects were heterogeneous in the ATBC Study, since certain analyses that demonstrated strong evidence of heterogeneity were published before their *JAMA* report^(9,10,117)^. Therefore, they should have considered whether it was appropriate to assume that there is one single uniform effect by vitamin E and all other antioxidants on total mortality.

Because of the pooling of diverse antioxidants and non-antioxidants, the calculated 5 % estimate of Bjelakovic *et al*. study is not valid. The review has been misleading readers about antioxidants for over a decade. There are several other meta-analyses that have similarly pooled different antioxidants into one single estimate of ‘antioxidant effect’^(118–124)^, and therefore the problem is not restricted to the Bjelakovic *et al*. study, although it remains the most highly cited study of its kind.

Several studies have indicated that supplementation of vitamin E^(9–17)^ and vitamin C^(18–21,125–132)^ might be beneficial in certain contexts. The clinical significance of such findings for these two major antioxidants is not clear. However, they should not be ignored on the basis that a meta-analysis erroneously claimed that all antioxidants increase mortality on average by 5 %^(1)^.

### Limitations of the study

The ATBC participants were all male smokers born before the Second World War. Thus, their childhood and youth were very different from those of later generations. In that respect, it is not evident how far various findings of the ATBC cohort can be extrapolated to the general current Western population. However, the background of this cohort does not compromise its use to examine the heterogeneity in the effects of BC supplementation in males since there is no reasonable basis to assume that the men of the ATBC Study were fundamentally different from all other men. Although the estimates of BC effects in the subgroups cannot be extrapolated to other groups of males, the observed heterogeneity provides no support for the concept that there is a uniform effect of BC for males.

The age of smoking initiation was one of the variables that defined the subgroup in which BC was harmful. Although many people may not recollect accurately their age of smoking initiation, this cannot generate the observed significant differences between the randomised groups. Errors in recalling smoking initiation would make the observed differences between the groups smaller.

The dietary intake of vitamins C and E, and fruit and vegetables were estimated at the baseline of the ATBC Study. Many participants may have changed their food consumption patterns over the years of the study. This can lead to misclassifications regarding the later parts of the follow-up. However, such misclassifications can attenuate the estimates of effect towards null, but cannot generate false heterogeneity between the randomised BC and placebo groups in the subgroups A to C.

The subgroup division in Table 3 was based on the determinations of vitamin C intake, and fruit and vegetable intake. It is therefore possible that these variables are not the actual modifiers of the effect of BC supplementation, but might instead correlate with some other actual modifying variables. If that were the case, however, it would not refute the strong evidence of heterogeneity in the BC effect over the three subgroups *per se* (Table 3.). The three subgroups would simply require redefining by relabelling them with the appropriate terms for the variables.

Finally, the dose of BC was particularly large as it increased the serum BC level 17-fold compared with the baseline levels. Evidently, we may expect much less harm from much lower doses of BC supplements even in the identified subgroups A and C.

### Future research directions

It has been estimated that over 30 billion dollars are expended per year on dietary supplements in the USA^(133)^. A substantial proportion of that 30 billion-dollar figure covers antioxidant supplements. The global market of BC, which includes its nutrient supplement sale sector, is also a billion-dollar business^(134,135)^. BC is available as a single nutrient supplement and it is also included in various multivitamin supplement combinations. Therefore, further research on the potential harms from BC is justified.

Subgroup analysis has often been discouraged because there have been numerous misleading findings that have resulted from using this approach. Biology is complex, however, and to assume that there is a single uniform treatment effect over the entire population seems implausible. Rather than simply calculating a single overall average effect, it may even be considered the ethical duty of the researcher to analyse large trials by subgroups in detail given the long-term commitment of study participants and the resources invested in such studies. Nevertheless, it is important to carry out subgroup analysis with caution and not over-interpret the findings.

### Conclusions

The primary goal of the present study was to investigate whether the effect of BC supplementation on total mortality in the ATBC Study cohort is heterogeneous. Strong evidence was found to indicate that the harm of BC supplementation is not uniform over the study population. This study also found that there is interaction between BC and vitamins C and E in humans. Nevertheless, the evidence of interaction was seen only in a small subgroup of 7 % of the ATBC Study participants, who already were a narrow selection of the general population as all were male smokers of age 50 years or over. Furthermore, the dose of BC was particularly high as it increased plasma BC level up to 17-fold. Thus, the observed interactions between BC and vitamins C and E should not be extrapolated to the general population. The strong evidence of heterogeneity in the BC effect on mortality found in this study is a further counterargument against study-level meta-analyses that have pooled different antioxidant trials into a single uniform estimate of effect. Smokers should avoid high doses of BC.

## Supplementary material

For supplementary material accompanying this paper visit http://dx.doi.org/10.1017/jns.2020.3. click here to view supplementary material
