# Supplementary material for: The effect of β-carotene on the mortality of male smokers is modified by smoking and by vitamins C and E: evidence against a uniform effect of nutrient
Source: J Nutr Sci. 2020 Mar 11;9:e11. doi: 10.1017/jns.2020.3 (PMC7082716; doi:10.1017/jns.2020.3)
Supplement: Supplementary file 1 [file S2048679020000038sup001.pdf]

The β-carotene effect on the mortality of male smokers is modified by smoking and by vitamins C and E: evidence against a uniform effect of nutrient

Harri Hemilä

Department of Public Health
University of Helsinki,
P. O. Box 20
FI-00014 University of Helsinki, Finland.
Email: harri.hemila@helsinki.fi

Supplementary file

submitted to Journal of Nutritional Science
https://www.cambridge.org/core/journals/journal-of-nutritional-science

ver 2019-11-29

Contents **Page**

Table S1: Comparison of baseline variables in groups A to C in the four ATBC Study arms 2

Table S2: No-vitamin E participants smoking 5-19 cigarettes per day: subgroup comparisons 3

Table S1: Comparison of baseline variables in subgroups A to C in the four ATBC Study arms

Variable	Placebo	BC	AT	AT+BC
**Group A**				
N	223	214	261	251
Age (y)	57.13	57.14	56.9	57.7
Weight (kg)	80	79	79	80
Cigarettes/d at baseline	30.6	30.0	30.5	30.0
Age of smoking initiation (y)	24.4	24.7	24.3	24.3
Vitamin C in food (mg/d)	64	63	63	65
β-carotene in food (mg/d)	1.49	1.32	1.41	1.53
Fruit (g/d)	47.5	47.4	44.6	48.1
Vegetables (g/d)	54.6	54.9	55.6	59.5
Alcohol (g/d)	20.4	23.0	25.8	21.1
**Group B**				
N	171	157	168	157
Age (y)	56.9	56.4	56.6	56.6
Weight (kg)	82	83	84	82
Cigarettes/d at baseline	30.0	29.3	29.0	30.8
Age of smoking initiation (y)	24.2	24.4	24.4	24.7
Vitamin C in food (mg/d)	121	117	119	121
β-carotene in food (mg/d)	2.35	2.40	2.42	2.49
Fruit (g/d)	91	85	91	97
Vegetables (g/d)	95	99	93	97
Alcohol (g/d)	20.0	20.6	17.4	21.0
**Group C**				
N	69	81	78	73
Age (y)	55.6	56.4	57.2	55.7
Weight (kg)	88.5	85.5	82.7	82.5
Cigarettes/d at baseline	30.6	30.1	30.8	30.2
Age of smoking initiation (y)	24.7	24.8	24.9	24.9
Vitamin C in food (mg/d)	170	176	169	184
β-carotene in food (mg/d)	3.45	3.64	3.14	3.89
Fruit (g/d)	215	213	211	247
Vegetables (g/d)	164	162	168	165
Alcohol (g/d)	18.3	21.2	20.7	21.0

Table S2: No-vitamin E participants smoking 5-19 cigarettes per day: subgroup comparisons

**Subgroup**	**Placebo arm**	**BC arm**
All		
RR (95% CI):	1.0 (ref.)	0.98 (0.84-1.15)
Deaths:	314	313
Participants:	2641	2668
**Vitamin C and fruit-vegetables**		
C: Vit C ≥90 mg/d and fruit-vegetables ≥275 g/d		
RR (95% CI):	1.0 (ref.)	0.89 (0.57-1.39)
Deaths:	40	37
Participants:	415	424
B: Vit C ≥90 mg/d and fruit-vegetables <275 g/d		
RR (95% CI):	1.0 (ref.)	0.92 (0.69-1.23)
Deaths:	98	92
Participants:	920	919
A: Vit C <90 mg/d		
RR (95% CI):	1.0 (ref.)	1.05 (0.84-1.32)
Deaths:	146	154
Participants:	1136	1137
**Weight**		
<80 kg		
RR (95% CI):	1.0 (ref.)	1.02 (0.84-1.25)
Deaths:	191	203
Participants:	1534	1582
≥80 kg		
RR (95% CI):	1.0 (ref.)	0.90 (0.69-1.16)
Deaths:	123	109
Participants:	1105	1083
**Alcohol**		
<14 g/d		
RR (95% CI):	1.0 (ref.)	0.91 (0.75-1.12)
Deaths:	192	176
Participants:	1659	1640
≥14 g/d		
RR (95% CI):	1.0 (ref.)	1.12 (0.84-1.48)
Deaths:	92	107
Participants:	812	840
